# Attitudes about Testicular Self-Examination among Polish Males

**DOI:** 10.3390/biology10030239

**Published:** 2021-03-19

**Authors:** Tomasz Milecki, Natalia Majchrzak, Adam Balcerek, Maciej Rembisz, Michał Kasperczak, Andrzej Antczak

**Affiliations:** 1 Department of Urology, Poznań University of Medical Sciences, 61-701 Poznań, Poland; mileckito@gmail.com (T.M.); a.j.balcerek@gmail.com (A.B.); maciek.rembisz@wp.pl (M.R.); aa26@poczta.onet.pl (A.A.); 2 Transplantology, General Surgery and Urology Department, Poznań District Hospital, 61-144 Poznań, Poland; drmajchrzak@gmail.com

**Keywords:** testicular self-examination, testicular cancer, cancer prevention, polish males

## Abstract

**Simple Summary:**

Testicular cancer (TC) affects men at a young age and has high survival rates. Most TCs are detected as palpable masses during self-examination (TSE) or physical examinations performed by General Practitioner (GP). The aim of the study was to discover the attitudes among Polish males regarding TSE and factors (environmental, social, educational) that affect intention to perform TSE. The mean age of the surveyed respondents was 32 years. Information about TC and how to perform TSE was obtained by 34.4% (*n* = 185) of the men. It was shown that the following factors increase men’s intention to perform TSE: TC in their family member (*p* < 0.05; HR = 5.9; 95% CI: 1.5–23.0), GP’s recommendations (*p* < 0.001; HR = 6.8; 95% CI: 3.2–14.3), concern expressed by their partner (*p* < 0.001; HR = 3.3; 95% CI: 2.1–5.3), and social campaigns (*p* < 0.001; HR = 2.6; 95% CI: 1.5–4.6). Approximately half of young polish males do not perform TSE. Access to information on TC prevention is limited. Teaching TSE will result in these men taking increased responsibility for their own healthcare.

**Abstract:**

Introduction: Epidemiological data indicate an increased incidence of testicular cancer (TC), making it the most common malignant tumor in men from aged 15–45. Oncological and urological associations recommend that men with specific TC risk factors should regularly perform a testicular self-exam (TSE). The aim of the study was to discover the attitudes among Polish males regarding TSE and factors (environmental, social, educational) that affect intention to perform TSE. Methods: An original survey containing 21 questions was used to conduct a study among the Polish branch of VW (Volkswagen Poland) employees. Results: A total of 522 fully completed questionnaires were collected. The mean age of the surveyed respondents was 32 years. Information about TC and how to perform TSE was obtained by 34.4% (*n* = 185) of the men. It was shown that the following factors increase men’s intention to perform TSE: TC in their family member (*p* < 0.05; HR = 5.9; 95% CI: 1.5–23.0), GP’s(General Practitioner) recommendations (*p* < 0.001; HR = 6.8; 95% CI: 3.2–14.3), concern expressed by their partner (*p* < 0.001; HR = 3.3; 95% CI: 2.1–5.3), and social campaigns (*p* < 0.001; HR = 2.6; 95% CI: 1.5–4.6). Conclusions: Approximately half of young polish males do not perform TSE. Access to information on TC prevention is limited. Further action is needed to improve men’s awareness of TC and TSE.

## 1. Introduction

TC (testicular cancer) is the most common male malignant neoplasm in the age group between 15–45 [1]. The incidence rate is 3–10 new cases per 100,000 men per year in Western Countries [2]. At diagnosis, 1–2% of cases are bilateral and the predominant histology is GCT (Germ Cell Tumor) (90–95% of cases) [2]. The most commonly diagnosed type of GCT are seminomas, accounting for about half of all GCT [3]. Known TC risk factors are the following: cryptorchidism, previous diagnosis of TC, genetic predispositions, and maternal estrogen exposure [4,5,6,7]. The most common symptom is a mass in the testis in the early period, but in many patients, there is widespread pain, swelling, or stiffness in the scrotum [8]. Acute testicular pain is less frequent and is more often caused by the testis’ swift expansion owing to intratumor hemorrhage or infarction [9].

Radical inguinal orchiectomy is the treatment of choice for men with suspected TC. The most important TC detection tool is the testicular examination performed by a doctor or by self-examination (TSE) [8]. TSE is an easy screening technique that involves inspection and palpation of the testes to detect any abnormalities. TSE is recommended to be done by men above 15 years of age once a month [10]. Regular TSE may allow early detection of TC. Diagnosis of TC in the early-stage may yield potential and measurable benefits to the patient, primarily contributing to a significant improvement in prognosis—when TC is diagnosed in the early stages, the rate of treatment is approximately 96%, and 80% for metastatic disease [11]. Moreover, early diagnosis of TC may avoid adjuvant treatment (radio and chemotherapy), i.e., therapeutic methods that may contribute to complications such as cardiotoxic and neurotoxic effects, and secondary cancers [4].

To date, no scientific research has been conducted that would indicate that the implementation of regular TSE as a screening in the male population contributes to oncological benefits. Therefore, according to the main oncological and urological associations (EAU and AUA) recommendations, only young men who are identified as having specific risk factors for TC should be particularly made aware of the need for regular TSE [12]. However, the majority of TC’s are detected by men as a result of TSE [5,13]. Moreover, performing TSE may be beneficial for men because it facilitates detection of diseases in the scrotum other than TC. Therefore, it is reasonable to educate young men about TC, the possibility of performing TSE, and take immediate medical consultation if any abnormalities in testis are found.

Knowledge of and awareness about TC and TSE among men world-wide is low [14,15,16,17]. It is proven that cancer-related awareness improvement requires multi-pronged measures, including primarily conducting campaigns and educational programs for the society and a proper training system for health care professionals [18]. Therefore, it is necessary to periodically evaluate the state of knowledge and awareness about particular diseases in society. Such information may be further used to correct any irregularities which may occur. The number of scientific reports on TC awareness in relation to the Polish population is still limited, therefore, we decided to make an up-to-date assessment.

The aim of the study was to discover the attitudes among Polish Males regarding TSE and factors (environmental, social, educational) that affect intention to perform TSE.

## 2. Materials and Methods

In 2019, a population study was carried out on 522 employees working at the Poznan Volkswagen branch. An original questionnaire was used consisting of 21 questions concerning demographic data and factors that may potentially affect preventive measures taken by men. Participation in the study was anonymous and voluntary. The exclusion criterion was a refusal of an employee to complete the questionnaire.

The statistical analysis was carried out with the use of the SPSS Statistics software. Descriptive statistics were carried out. Logistic regression was used as both univariate and multivariate to predict the role of a variable on intention to perform TSE respectively. Findings with a *p*-value < 0.05 at a 95% confidence interval were considered as statistically significant. The results are presented in tables and as a pie chart.

The study was approved by the Bioethics Committee of the Poznań University of Medical Sciences (opinion no. 108/19). Written consents of the respondents and the director of the company were also obtained.

## 3. Results

### 3.1. Socio-Demographic Characteristics of the Study Participants

In this study, a total of 522 subjects participated (Table 1). The mean age of the respondents was 32 years (± 9.9 SD, range 18–59). A total of 30.3% (*n* = 158) of the respondents lived in rural areas, 13.2% (*n* = 69) lived in towns with less than 10,000 inhabitants, 33.7% (*n* = 176) lived in medium-sized towns with 10,000 to 100,000 inhabitants, and 22.8% (*n* = 119) of the respondents lived in cities with over 100,000 inhabitants. The most of all, i.e., 44.8% (*n* = 234), of the respondents graduated from a secondary school, 24.9% (*n* = 130) graduated from a vocational school, and 26.2% (*n* = 137) had university-level education. White-collar employees comprised 26.6% (*n* = 139) of the respondents. The marital status of the respondents was as follows: single 32.8% (*n* = 171), in an informal relationship 9.8% (*n* = 51), and married 57.3% (*n* = 299). A total of 58.8% (*n* = 307) of the respondents had offspring and 88.1% (*n* = 460) had siblings. The educational background of the mother and father of the surveyed employees was as follows: primary school, 5.9% (*n* = 31) and 3.8% (*n* = 20); secondary education, 39.3% (*n* = 205) and 32.6% (*n* = 171); vocational education, 40.6% (*n* = 212) and 52.1% (*n* = 272); and higher education, 14.2% (*n* = 74) and 11.3% (*n* = 59), respectively. A total of 20.3% (*n* = 106) of the respondents declared that they had a family member with medical education. Health was important for 43.9% (*n* = 229) of the respondents and very important for 55.4% (*n* = 289). Moreover, most of the respondents considered their appearance to be important 64.4% (*n* = 336) or very important 32.2% (*n* = 168). It was found that 29.9% (*n* = 156) of the respondents practiced sport two or three times a week; 23.2% (*n* = 121) practiced sport once a week; 8.2% (*n* = 43) practiced sport more frequently, and most of the respondents, i.e., 38.7% (*n* = 202), engaged in physical activity less than once a week.

### 3.2. The Practice of TSE among Respondents

**Figure 1:**
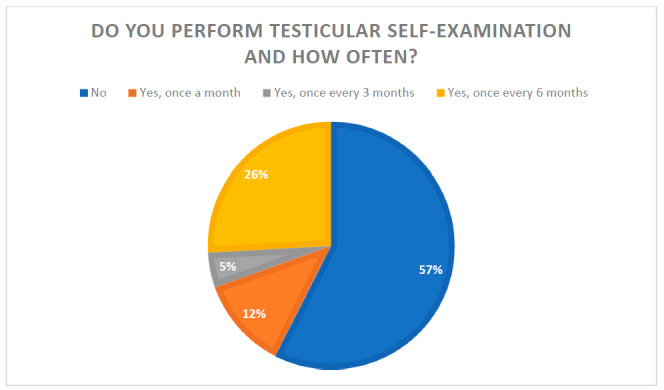
The results of respondents’ answers to the question “Do you perform testicular self-examination and how often?”.

It was demonstrated that 57.5% (*n* = 300) of men in Poland did not perform TSE at all. Among those who did, 28.4% (*n* = 63) declared that they performed TSE once a month, 10.8% (*n* = 24) once every 3 months, while the highest percentage 60.8% (*n* = 135) performed TSE once every 6 months (Figure 1).

### 3.3. Source of Knowledge about TSE

Information and instructions on how to perform TSE were obtained by 34.4% (*n* = 185) of the men (Table 1). Most often this information was provided in the form of medical consultation, 32.4% (*n* = 60); social campaign, 42.2% (*n* = 78); or from their partner, 62.2% (*n* = 115). In total, 84.9% (*n* = 443) of men would apply medical consultation immediately after detecting a testicular lesion. The majority of the respondents, i.e., 59.6% (*n* = 311), and 36.8% (*n* = 192) would apply medical consultation from a urologist and GP respectively.

### 3.4. Factors Associated with Practice of TSE

Table 1 represents the results of univariate and multivariate logistic regression, which demonstrated the impact of specific factors (socio—demographic data) to the practice of TSE among Polish males. Overall, 57.5% (*n* = 300) of respondents do not perform TSE at all and 42.5% (*n* = 222) perform TSE. Using a univariate logistic regression model, we demonstrated that following factors: occurrence of TC in the family member (HR = 5.7; 95% CI: 1.6–20.2; *p* < 0.05), recommendations from a GP (HR = 8.4; 95% CI: 4.2–17.1; *p* < 0.001), concern expressed by their partner (HR = 4.1; 95% CI: 2.6–6.3; *p* < 0.001) and social campaigns (HR = 3.9; 95% CI: 2.3–6.7; *p* < 0.001), and care about health (HR = 1.6; 95% CI; 1.1–2.3; *p* < 0.01) increase the probability of practice of TSE among respondents. The final multivariate logistic regression model demonstrated that the following factors have an independent influence on whether men undertake TSE: occurrence of TC in the family (HR = 5.9; 95% CI: 1.5–23.0; *p* < 0.05), recommendations from a GP (HR = 6.8; 95% CI: 3.2–14.3; *p* < 0.001), concern expressed by their partner (HR = 3.3; 95% CI: 2.1–5.3; *p* < 0.001) and social campaigns (HR = 2.6; 95% CI: 1.5–4.6; *p* < 0.001), and care about health (HR = 1.6; 95% CI; 1.1–2.5; *p* < 0.01).

## 4. Discussion

The study performed by our research team is an analysis that assesses TSE practice among Polish males with respect to socio-demographic aspects. First of all, our study demonstrated that 57.5% of Polish males aged 18–59 did not perform TSE at all and only 25% performed regular TSE at least once a month. Moreover, our study has shown a significant improvement in TSE practice among men who had TC in their family members, who received GP’s recommendations, whose partner expressed concern about this issue, or who attend social campaigns about TC.

Our results are to a large extent consistent with that of other research teams. First of all, it is worth nothing the results of a study of Polish men published in 2018 [14]. The study was conducted on a group of 204 students of a medical university. It showed that men’s awareness of TSE and the availability of information in this scope were low. According to the authors, 59% of men in Poland do not examine their testicles at all. Only 21% perform such examination as frequently as recommended, i.e., once a month. What is more, half of the people who regularly examine their testicles are not sure whether their examination is reliable, which results from the lack of theoretical and practical knowledge. These authors reported that the main source of knowledge for the respondents was school and media (46%), while only 11% of them received recommendations from their GP.

Similar findings were also reported in a second Polish study from 2020 where TSE practice and TC knowledge among high school students and medical university students was assessed [15]. Authors of this paper found that medical students have a higher level of knowledge related to TC than high school students. This study, similar to a previous one, confirmed that level of education had a significant impact on TSE practice and TC awareness among respondents. It was found that most high school students (80%) and half of medical students (50%) have never performed TSE. Moreover, only 30% of medical university students and 9.9% of high school students performed TSE regularly, at least once a month. This study highlighted that higher level of knowledge among participants was correlated with higher awareness of TC and more frequent TSE practice. Analogous results were confirmed in a German population study where authors also found that medical students had a significantly higher TC knowledge than non-medical students [16]. In addition, medical students conducted TSE more frequently (34.7%) than non-medical students (18.8%). As a result, both studies have proven that even in the group of medical students the level of knowledge and awareness about TC and TSE was better than in the general population, but it was still not sufficient and medical personnel may have some gaps of knowledge regarding TC.

Similar conclusions may also be drawn from other publications originating from culturally different regions of the world. For example, a survey conducted on a group of 174 men (nursing and dietetics students) in Turkey also showed that about 80% of the respondents were not aware of the possibility of performing TSE [17]. The authors of the publication referred to above showed that the respondents’ level of knowledge about TC, prevention methods, and details of TSE was low. Their project included lectures for the respondents, followed by a knowledge check after 3 months, which showed a significant improvement and increase in awareness of the possibility of TSE. Our study did not assess the state of knowledge of the respondents, but indirectly showed that people who received appropriate recommendations in this respect, e.g., from their GP, or obtained information from social campaigns were much more likely to perform TSE. The above hypothesis was studied in other works, which also showed that an increase in the level of knowledge translates into an increase in the level of awareness of TSE [19,20,21].

An improvement in the level of knowledge among the society in a given area of medicine may be achieved by launching an information campaign. An example of a worldwide campaign on urological diseases in men is the “Movember campaign”. It aims to raise social awareness of men’s health problems, especially prostate cancer and TC. The campaign was launched in 2004 in Australia and New Zealand and has been continued in Poland since 2012 [22]. In our survey, we showed that the annual “Movember campaign”, which has continued for many years, reached only 15% of the surveyed men in Poland. Moreover, we showed that although such campaigns had a positive impact on men’s awareness, it was still significantly lower compared to recommendations made by a GP. A recently published study based on the analysis of data from Google showed that despite many years of efforts, the “Movember campaign” is not directly identified with testicular or prostate cancer [23,24]. Similar conclusions were drawn in another study, also based on data from Google, which showed that the social campaign on breast cancer (“Pink October”) is more popular on the Internet than the campaign on TC and prostate cancer (“Movember campaign”) [25]. The results of the studies referred to above, as well as ours, may lead to a common conclusion as to the limited effectiveness of the “Movember campaign” due to the lack of a clear message and limited coverage, in particular in social media.

Our study demonstrated also that a partner’s concern for their partner’s health is an important factor influencing men’s awareness and willingness to undertake TSE. Men who have this problem raised by their partners are more likely to undertake TSE. Additionally we found that the TSE and TC issue was raised by women (partner) more often than by a GP. The reason for this may be that regular urological check-ups in young males are not recommended by oncological scientific associations. Moreover, it is important to underline that TSE by men is a taboo subject and men may reject medical consultation [26]. It was also shown that men were also reluctant to seek medical care and were reluctant to admit to ailments, which may delay the diagnosis. Available studies indicate that women attach more importance to their own health and that of other family members than men [27,28]. Previous studies have shown that one partner’s attitudes and behavior are associated with the other partner’s intention in the context of cancer and health care screening [26,29]. To summarize, TC-educated women could act as a motivator for TSE or could also actively perform the testicular examination. The above hypothesis was confirmed in our study and also in the studies performed by Vallo et al. and Braga et al. [16,30]. Therefore, we think that multi-pronged efforts of the medical community to prevent malignant tumors of male organs should also take into account the female gender.

In our study, the relationship between socio-demographic factors and TC awareness and TSE practice was not confirmed. However, we found that men with positive family history of TC had higher awareness of TC and TSE. The higher awareness of TC in the group of men with positive family history of TC was confirmed also in other studies [15,31]. Family history of TC is not a modifiable risk factor predisposing for TC development (four to nine times greater risk) and for this particular group of men, EAU and NCCN recommend regular TSE [32]. Nevertheless, there are also others significant factors for TC development like cryptorchidism, testicular atrophy, or testicular micro-calcifications. In our study, unfortunately we found that men with positive history of other testicular diseases were not aware of TC and TSE. This finding indicates that Polish males may have particular gaps of knowledge regarding TC risk factors. Similar conclusions were found in an already mentioned Polish study where unsatisfying awareness of the link between TC and cryptorchidism was reported among men [15].

Our study allowed us to draw some important conclusions, which are in line with the results available in the literature. Moreover, this is one of few studies in this respect concerning the population of Central and Eastern Europe. What is important is that it covered a numerous group, which was diverse in terms of demographic and social aspects. However, our study was not without some shortcomings. The most important was that it was based entirely on data obtained from a single center. Therefore, an exceptionally homogeneous group in terms of religion and culture was analyzed. These factors may certainly play a role in cancer prevention, especially with regard to genitals, whose examination is a taboo subject. However, we hope that extrapolation from a group as large as this one can provide the basis for drawing conclusions for society as a whole and also for other parts of the world.

## 5. Conclusions

Improvement of cancer-related awareness requires a multi-pronged measures, including primarily conducting campaigns and educational programs for the society and a proper training system for health care professionals. TSE is a simple screening technique that may allow early detection of TC. Our study confirmed that recommendations from a GP, information campaigns, concern expressed by their partner, and TC in the family increase the probability of men undertaking TSE. Unfortunately our study revealed that about half of Polish males do not perform TSE, indicating that level of awareness of TC among Polish males may be on a low level. Moreover, we found that access to TC/TSE information in Poland is also limited, and that is why further action is needed to improve this matter. Social campaigns and GPs should encourage more men to perform TSE. We suggest that TC education should also target women because TC-educated women could act as a motivator for male self-examination.

## Figures and Tables

**Table 1:** Factors associated with practice of TSE among respondents (univariate and multivariate regression model); NA—not applicable in the final model.

Factors	Results	Univariate HR (95% CI)	Multivariate HR (95% CI)
Age	0- <20 years 36 (6.9%) 1- 20–29 years 145 (27.8%) 2- 30–39 years 180 (34.5%) 3- 40–49 years 126 (24.1%) 4- 50–59 years 35 (6.2%)	1 (reference) 0.6 (0.3–1.3) 0.6 (0.3–1.4) 0.7 (0.3–1.5) 1.1 (0.4–2.7)	NA
Place of residence (population)	0- Village 158 (30.3%) 1- Below 10,000 69 (13.2%) 2- 10,000 to 100,000 176 (33.7%) 3- Above 100,000 119 (22.8%)	1 (reference) 1.4 (0.8–2.4) 1.1 (0.7–1.7) 0.9 (0.5–1.4)	
Education	0- Primary school 21 (4%) 1- Secondary school 234 (44.8%) 2- Vocational school 130 (24.9%) 3- University 137 (26.2%)	1 (reference) 0.6 (0.2–1.5) 0.6 (0.2–1.5) 0.4 (0.2–1.1)	
Profession	0- Secondary school student 30 (5.7%) 1- University student 9 (1.7%) 2- Blue-collar worker 344 (65.9%) 3- White-collar worker 139 (26.6%)	1 (reference) 0.4 (0.1–1.8) 0.5 (0.3–1.1) 0.6 (0.3–1.3)	
Sport (frequency)	0- Less than once a week 202 (38.7%) 1- Once a week 121 (23.2%) 2- 2 or 3 times a week 156 (29.9%) 3- Every day 43 (8.2%)	1 (reference) 1.1 (0.7–1.7) 1.3 (0.9–2.0) 1.6 (0.8–3.2)	
My health is for me	0- Important 233 (44.6%) 1- Very important 289 (55.4%) 2- Not important 0 (0%)	1 (reference) 1.6 (1.1–2.3) -	1.6 (1.1–2.5)
My appearance is for me	0- Not important 18 (3.4%) 1- Important 336 (64.4%) 2- Very important 168 (32.2%)	1 (reference) 1.0 (0.4–2.7) 1.5 (0.6–4.0)	NA
Marital status	0- Single 171 (32.6%) 1- Informal relationship 51 (9.8%) 2- Married 299 (57.3%)	1 (reference) 1.5 (0.8–2.8) 1.0 (0.7–1.5)	
Children	0- No 215 (41.2%) 1- Yes 307 (58.8%)	1 (reference) 0.9 (0.6–1.3)	
Siblings	0- No 62 (11.9%) 1- Yes 460 (88.1%)	1 (reference) 0.7 (0.4–1.3)	
Mother’s education	0- Primary school 31 (5.9%) 1- Secondary school 205 (39.3%) 2- Vocational school 212 (40.6%) 3- University 74 (14.2%)	1 (reference) 0.9 (0.4–1.8) 0.9 (0.4–1.9) 1.0 (0.4–2.4)	
Father’s education	0- Primary school 20 (3.8%) 1- Secondary school1 171 (32.6%) 2- Vocational school 272 (52.1%) 3- University 59 (11.3%)	1 (reference) 0.7 (0.3–1.7) 0.8 (0.3–1.9) 0.8 (0.3–2.3)	
A family member with medical education	0- No 416 (79.7%) 1- Yes 108 (20.3%)	1 (reference) 1.2 (0.8–1.9)	
Have you ever had any testicular disease?	0- No 495 (94.8%) 1- Yes 27 (5.2%)	1 (reference) 2.0 (0.9–4.5)	
TC in your family	0- No 507 (97.2%) 1- Yes 15 (2.8%)	1 (reference) 5.7 (1.6–20.2)	5.9 (1.5–23.0)
Has your GP informed you about the possibility to perform TSE?	0- No 462 (88.5%) 1- Yes 60 (11.5%)	1 (reference) 8.4 (4.2–17.1)	6.8 (3.2–14.3)
Does your partner raised the problem of TC or TSE?	0- No 407 (78%) 1- Yes 115 (22%)	1 (reference) 4.1 (2.6–6.3)	3.3 (2.1–5.3)
Do you know any social campaigns on TC?	0- No 444 (85%) 1- Yes 78 (15%)	1 (reference) 3.9 (2.3–6.7)	2.6 (1.5–4.6)

## Data Availability

The data presented in this study are available on request from the Tomasz Milecki (project manager). The data are not publicly available due to polish law and law at the Medical University of Poznań.
